# Poster Session II - A298 LONG TERM OUTCOMES IN SMALL BOWEL CROHN’S DISEASE STRICTURES WITH PRE-STENOTIC DILATION ON INTESTINAL ULTRASOUND

**DOI:** 10.1093/jcag/gwaf042.298

**Published:** 2026-02-13

**Authors:** R Reji, M O’Brien, R Rosentreter, A AlDarwish, J Besney, R Ingram, G G Kaplan, C Ma, C Seow, J St-Pierre, K Novak, R Panaccione, F Rieder, C Lu

**Affiliations:** Division of Gastroenterology and Hepatology, University of Calgary, Calgary, AB, Canada; Division of Gastroenterology and Hepatology, University of Calgary, Calgary, AB, Canada; Division of Gastroenterology and Hepatology, University of Calgary, Calgary, AB, Canada; Division of Gastroenterology and Hepatology, University of Calgary, Calgary, AB, Canada; Division of Gastroenterology and Hepatology, University of Calgary, Calgary, AB, Canada; Division of Gastroenterology and Hepatology, University of Calgary, Calgary, AB, Canada; Division of Gastroenterology and Hepatology, University of Calgary, Calgary, AB, Canada; Division of Gastroenterology and Hepatology, University of Calgary, Calgary, AB, Canada; Division of Gastroenterology and Hepatology, University of Calgary, Calgary, AB, Canada; Division of Gastroenterology and Hepatology, University of Calgary, Calgary, AB, Canada; Division of Gastroenterology and Hepatology, University of Calgary, Calgary, AB, Canada; Division of Gastroenterology and Hepatology, University of Calgary, Calgary, AB, Canada; Cleveland Clinic Lerner Research Institute, Cleveland, OH; Medicine, University of Calgary, Calgary, AB, Canada

## Abstract

**Background:**

Small bowel Crohn’s disease (CD) stricture severity is evaluated by magnetic resonance enterography (MRE), computed tomography enterography (CTE) or intestinal ultrasound (IUS) with comparable accuracy. Prestenotic dilatation (PSD) size on MRE and CTE is associated with increased surgical risk. No recent studies have evaluated the impact of PSD size in small bowel CD strictures on IUS and long-term outcomes.

**Aims:**

To describe the proportion of patients with small bowel CD strictures on index IUS and subsequent CD-related hospitalization or surgery.

**Methods:**

Retrospective chart review from a single tertiary center was undertaken to identify patients with small bowel CD strictures and first evidence of PSD documented on IUS. Patients were excluded if PSD was identified on MRE/CTE prior to index IUS. Unpaired t-test with Welch’s correction and Fisher’s exact test assessed continuous and categorical variables, respectively.

**Results:**

79 CD patients with PSD on index IUS (2014-2025) with 4.8 years of median follow up (1-17.5) were identified. Of these, 57.0% (n = 45) had naïve and 43.0% (n = 34) had anastomotic strictures. Mean CD duration at PSD detection was 12 years (0-55). Median BWT was 7.0 mm (3-12), lumen size 2.0 mm (0.7 – 5), PSD size 2.5cm (1.4-8.0) and stricture length 10.0cm (2-50).

From PSD detection, 46.8% (37) required stricture surgery within median of 13 months (0-115) and 26.5% (21) needed hospitalization for CD within median of 7 months (0-86). Larger PSD size (mean 3.0 cm vs 2.4 cm; p = 0.01) predicted need for surgery (p = 0.01) but not hospitalization, while corticosteroid use was associated with both (p = 0.04).

**Conclusions:**

Nearly 50% of patients with small bowel CD strictures and PSD needed surgery within median 13 months. PSD size and steroid use predicted progression, indicating IUS can aid in prognosis and therapy timing

**Funding Agencies:**

None

**A298 Table 1:** Outcomes

OUTCOMES	n (%)
CD Hospitalisation	21(27%)
Median time from PSD diagnosis on IUS to hospitalisation (m)	7 (0-86)
CD surgery	37 (47%)
Median time from PSD diagnosis on IUS to surgery (m)	13 (0-115)
Steroid use	37 (47%)
Endoscopic balloon dilatation	11 (14%)

